# Late-Night Digital Media Use in Relation to Chronotype, Sleep and Tiredness on School Days in Adolescence

**DOI:** 10.1007/s10964-022-01703-4

**Published:** 2022-11-19

**Authors:** Laura Kortesoja, Mari-Pauliina Vainikainen, Risto Hotulainen, Ilona Merikanto

**Affiliations:** 1 Centre for Educational Assessment, University of Helsinki, 00014 Helsinki, Finland; 2 Faculty of Education and Culture, Tampere University, 33014 Tampere, Finland; 3 Department of Psychology and Logopedics and SleepWell Research Program, Faculty of Medicine, University of Helsinki, Helsinki, Finland; 4 Finnish Institute for Health and Welfare, Helsinki, Finland; 5 Orton Orthopaedics Hospital, Helsinki, Finland

**Keywords:** Diurnal preference, Eveningness, Screentime, Insufficient sleep, Sleep quality, Youth

## Abstract

Previous studies on late-night digital media use and adolescent sleep have not considered how chronotype, a natural tendency to be awake or asleep at certain time, is associated with this relationship. Therefore, the nature of the relationship between late-night digital media use and sleep in different chronotypes remains still unknown. The sample consisted of 15–20-year-old Finnish adolescents (*n* = 1084, mean age = 16.9 years, SD = 0.93, 45.7% female). This study examined whether chronotype, measured as diurnal type and midpoint of sleep, was associated with the time of evening/night when digital media was used. Associations between the use of different forms of digital media and sleep quality, sleep duration and tiredness on school days were also investigated. Finally, the mediation effect of late-night digital media use to the relationship between chronotype and sleep was examined. Generalized linear models showed that evening chronotype, weekend midpoint of sleep, and the time of evening or night at which digital media was used were associated with more insufficient sleep and tiredness, lower sleep quality and shorter sleep duration on school days. The total use of all media forms, i.e., late-night digital media for music, movies/series, social media, and studying, were associated with shorter sleep duration and more insufficient sleep and daytime tiredness. Late-night social media use also mediated the association between diurnal type and sleep quality. Watching movies or listening to music late at night was the strongest mediator of the association between diurnal type and sleep and tiredness. The most prominent finding shows that of the all different media forms, watching movies or listening to music late at night were associated with increased daytime tiredness, whereas late social media use was associated with poor sleep quality. These interactions were pronounced especially for evening-types. The findings of the current study suggest that the negative effects of late-night media use are reflected especially in sleep quality and daytime tiredness among evening-types during adolescence.

## Introduction

Digital media is omnipresent in our society. Media plays a key role in the lives of children and adolescents in particular, as they have grown up with digital media (Prensky, 2001). The use of different screens is also becoming a common bedtime routine for children and adolescents. It delays the time of going to sleep (Bartel et al., 2015) and continues even after lights are switched off for sleep (Rafique et al., 2020). Late-night digital media use affects adolescents’ sleep through different mechanisms, such as exposure to blue light (Crowley et al., 2015) and the emotional aspects of engaging with social media (Scott & Woods, 2019). Previous studies have suggested that social media use is also associated with delayed bedtime (Harbard et al., 2016) and shorter sleep duration in adolescence (Hamilton et al., 2020).

Insufficient sleep is a serious concern, as almost one-third of children and adolescents do not get as much sleep as is recommended (Chaput et al., 2016). Poor sleep quality is also highly prevalent among adolescents (Michaud & Chaput, 2016). The implications of continuous sleep deprivation extend to all areas of life, weakening learning and performance (Kronholm et al., 2015), and lowering the quality of life (Reidy et al., 2016) and well-being (Mireku et al., 2019). Considering what crucial periods childhood and adolescence are for brain development, sleep problems are a serious concern (Giedd et al., 1999). Insufficient sleep is related to poor executive functioning such as cognitive abilities, which are crucial for learning (Kuula et al., 2015) and academic performance (Hysing et al., 2016). Although digital media use has been found to have a causal effect on adolescents’ sleep restricting time asleep (Poulain et al., 2019), further research is needed of the underlying mechanisms, such as media content or time displacement (Hale et al., 2019). The current study aimed at investigating the relationship between different forms of late-night digital media use and sleep among different chronotypes in adolescence.

Chronotype, which refers to a biological trait of individual circadian timing of physiological and behavioural functions (Czeisler & Gooley, 2007; Merikanto et al., 2021), regulates sleep timing from childhood to late adolescence. The onset of puberty brings a shift towards eveningness, occurring as, for example, later sleep-wake schedules during adolescence (Merikanto et al., 2018). Morningness starts to decrease already around the age of 12, and continues into late-adolescence/early adulthood, when eveningness reaches its peak (Roenneberg et al., 2007). Especially for evening-types, this means that the desired bedtime on school evenings may be too early for their innate circadian rhythm (Estevan et al., 2018). Evening media use has also increased among the adolescent population. Using media before going to sleep can postpone bedtime even further and delay adolescents’ sleep onset for longer. (Hysing et al., 2015.) As a result, adolescents might not be physically capable of making themselves sleep as early as desired and may eventually accumulate chronic sleep debt (Åkerstedt et al., 2010), especially those with a higher tendency for eveningness (Merikanto et al., 2018).

There is a paucity of research on different forms of digital media use and their associations with well-being (e.g., sleep and alertness), and the potential mediating variables (Guerrero et al., 2019). Although some research has been carried out on late-night digital media use and how it affects sleep (Tarokh et al., 2019) and daytime tiredness (van der Schuur et al., 2019), the associations between different forms of digital media (e.g., social media) and sleep remain relatively unknown (Scott & Woods, 2019). Knowledge of how diurnal preference is related to late-night digital media use and sleep is still lacking. Further, the effect of different types of media content before bedtime and their associations with sleep remain unknown. As chronotype is rather a stable state (Koskenvuo et al., 2007) and has a genetic basis (Jones et al., 2019), in the form of genetic variants (Merikanto et al., 2021) it is more likely to explain the time of the day when media is used than vice versa (Lin et al., 2021). Therefore, information on whether late-night digital media use mediates the association between chronotype and sleep deprivation is much needed.

## Current Study

The association between late-night digital media use and sleep for different chronotypes is not yet known. The present study aimed to investigate how the use of different forms of late-night digital media associate with chronotype and sleep, and to mediate the relation between diurnal preference and sleep in adolescence using general linear models and mediation analyses. In light of previous longitudinal and cohort studies, it is expected that chronotype would regulate the timing of late-night use of digital media that late-night use of digital media would restrict time asleep and impair sleep quality among evening-types in particular and that it would be associated with tiredness. It is also hypothesized that the mediation effects of different forms of late-night digital media use would vary from one to another in the association between chronotype and sleep duration, sleep quality, insufficient sleep, and tiredness.

## Methods

### Participants and Procedure

Cross-sectional data were gathered from 1127 Finnish students in general upper secondary education and vocational education and training, their age varying between 15 and 20 (mean age = 16.9, SD = 0.93, 45.7% female). This was done anonymously using paper-and-pencil questionnaires in 2016 as part of normal schoolwork. The participants were recruited from different high schools and vocational schools around Finland. Participation was voluntary. Information was only collected from students who were present during the school day. Data were only collected from Tuesday to Thursday during ordinary school weeks, and unconnected to academic year holidays, from 2016 to 2017. About 1145 participants produced eligible answers, but the responses of students who were older than 20 were omitted. The total analytical sample of this study consisted of 1084 adolescents aged 15–20 and provided information on their chronotype, late-night digital media use, and sleep.

### Measures

#### Chronotype assessment

This study examined chronotype from the perspectives of diurnal preference and habitual sleep-wake rhythm assessed as the weekend midpoint of sleep. Diurnal preference was assessed using the Morningness-Eveningness Questionnaire, which is a self-assessment questionnaire for determining morningness and eveningness in human circadian rhythms (Horne & Östberg, 1976). Diurnal preference was measured with a commonly used shortened six-item version (consisting of items 4, 7, 9, 15, 17 and 19 from the original MEQ, Cronbach’s *α* = 0.7) of this questionnaire (the MEQr) (Hätönen et al., 2008). The MEQr is a suitable psychometric tool for measuring chronotype at a population-based level (Merikanto et al., 2012) and among adolescents (Merikanto et al., 2017) and it explains 83% of the total variation of the original full MEQ (Hätönen et al., 2008). The sum score of MEQr ranges from 5 to 27, and categorical classes are formed from the total score of the scale as follows: Definitely/Moderately Evening Type < 12; Intermediate Type: 12–17; Definitely/Moderately Morning Type: >17. In this study, the categorical classes were used in the generalized linear models, and the total score in the mediation analyses.

Weekend sleep midpoint was calculated from the self-reported weekend bedtimes and wake-up times and used it to measure the phase of entrainment (Roenneberg et al., 2003). Weekend bedtimes and wake-up times were elicited by two questions: ‘When do you usually go to bed on Friday and Saturday nights?’ and ‘When do you usually wake up on Saturday and Sunday mornings?’. Weekend sleep duration was calculated as the hours between weekend bedtime and wake-up time. Weekend sleep midpoint was then determined from the weekend sleep duration as half of the time spent asleep.

#### PSQI sleep quality

Sleep quality was assessed using the 15-item version of The Pittsburgh Sleep Quality Index, which evaluates subjective sleep quality over the last 30 days (Buysse et al., 1989). The PSQI is translated into 48 languages and has been used in a wide range of population-based and clinical studies. The suitability of the PSQI for clinical and psychometric use has been evaluated by several researchers and its validity is supported by similar findings regarding sleep quality in groups using the PSQI or sleep measures with polysomnography (Buysse et al., 2008). The PSQI is also widely used to measure sleep quality among adolescents (de la Vega et al., 2015). Its 19 items are scored from 0 to 3. Only the self-reported items rated 0–3 were used for the analyses. The total sum score range was from 0 to 21, and a higher sum score, indicating worse sleep quality, was used as a measure of subjective sleep quality.

#### Bedtime, wake-up time, and sleep duration on school days

Participants self-reported their bedtime and wake-up time during the school week. Bedtime on school days was elicited by asking: ‘When did you go to bed yesterday?’ Wake-up time was elicited by the question: ‘When did you wake up today?’. The response scales consisted of self-reported hours and minutes for bedtime and wake-up time. To obtain the sleep information for school days, data were only collected from Tuesday to Thursday during ordinary school weeks. Sleep duration on school days was calculated as the hours between the self-reported bedtime on the previous school day and the wake-up time on the following school day.

#### Insufficient sleep on school days

The adolescents assessed whether their sleep was insufficient during the last five school days by answering an open question ‘Think about the last five school days. On how many of these days did you feel you had not slept enough when you woke up in the morning?’. The adolescents reported the number of school days on which they felt they had not got enough sleep during the last five school days.

#### Tiredness on school days

Tiredness on school days was measured by asking two questions: ‘How tired do you feel on school mornings?’ and ‘How tired do you feel on school days?’. The adolescents assessed their tiredness on school days using a five-point Likert scale, on which 1 = very tired and 5 = very alert. The sum score of these two items was used as a measure of tiredness on school days (Cronbach’s *α* = 0.6).

#### Evening and late-night digital media use during school week

The average of daily evening and late-night digital media use was assessed by a multiple-choice question developed for this study: ‘Which of the following things do you usually do when using digital media/the internet in the evening/night during a school week?’. The five different forms of digital media use that were assessed were social media, movies or music, studying, gaming and hobbies. The total use of all media forms (Cronbach’s *α* = 0.8) was formed by summarizing the scores based on social media use, watching movies/series or listening to music, studying, gaming, and hobbies. Social media was defined as updating profiles, messaging, chatting, following friends online, writing or commenting on blogs, taking part in debates, or making statements online. Watching movies/series or listening to music was defined as listening to music or watching movies or series online. Studying was defined as study-related activities online. Gaming was described as gaming to improve one’s gaming skills, developing games or gaming for fun. Hobbies were defined as other than gaming-related hobbies, for example, coding, making movies, videos, artwork, handicrafts, or other hobbies online. The question had eight response options: before 9 pm, from 9 to 10 pm, from 10 to 11 pm, from 11 to 12 pm, from 12 pm to 1 am, from 1 to 2 am, from 2 to 3 am, and after 3 am. If the respondent chose several time windows for their digital media use, digital media use time was used to estimate late-night digital media use. To form equal group sizes, the last categories of digital media use were merged. For the total use of all media forms – social media use, watching movies/series or listening to music and gaming, categories ‘from 1 am to 2 am’, ‘from 2 am to 3 am’ and ‘after 3 am’ were combined to form a new category, labelled ‘after 1 pm’. For studying and hobbies, categories ‘from 11 to 12 pm’, ‘from 12 pm to 1 am’, ‘from 1 to 2 am’, ‘from 2 to 3 am’ and ‘after 3 am’ were combined to form a new category, labelled ‘after 11 pm’.

#### Digital media use after lights out

Digital media use after lights out was elicited by asking ‘For how long do you still use your phone, smartphone, computer, tablet, TV, radio, or game console in your bedroom after you have switched lights off for sleep?’. The six response options were: Not at all, 0–10 min, 10–20 min, 20–60 min, 1–2 h, and more than 2 h.

#### Detriments of digital media use

The adolescents assessed the negative effects of digital media use on smartphones, computers, and tablets by answering the following four questions: 1. ‘I don’t have enough time for anything other than the internet.’ 2. ‘I don’t sleep enough because I use the internet too much.’, 3. ‘My sleep quality is poor because it is affected by my internet use in the evening or at night.’, 4. ‘I think I am addicted to the internet’. The five response options were: Totally disagree, disagree, neither agree nor disagree, agree, totally agree. The sum score of these questions was used as a subjective measure of the negative effects of digital media use (Cronbach’s *α* = 0.8).

### Data Analyses

First, one-way ANOVA tests were conducted for continuous dependent variables to compare differences in terms of sleep duration, sleep quality, insufficient sleep, and tiredness. Chi-square tests were used for the ordinal dependent variables to compare differences in terms of the latest time for different forms of late-night media use by diurnal type. Using generalized linear models, associations between chronotype and late-night digital media use and late-night digital media use and sleep duration, sleep quality, insufficient sleep, and tiredness on school days were analysed. Finally, using mediation analyses, the mediation effect of late-night digital media use mediated on the association between chronotype and sleep duration, sleep quality, insufficient sleep, and tiredness on school days was examined. As diurnal preference was more strongly associated with late-night digital media use than the midpoint of sleep at the weekend, sleep duration, sleep quality, insufficient sleep, or tiredness on school days, it was used to measure chronotype in the mediation analyses. Mediation analyses were only conducted on the forms of digital media use that were significantly associated with sleep duration, sleep quality, insufficient sleep, and tiredness. Process Macro (Hayes*,* 2018) was used in linear regression models and 5000 bootstrapping with bias-corrected Confidence Intervals (CI). All regression analyses were adjusted for gender and age. Evening media use before 9 pm was used as the reference group in the analyses. All analyses were performed using IBM SPSS (SPSS Inc., version 24.0).

## Results

### Sleep and Tiredness during School Week by Diurnal Type

Of the analytical sample, 12.9% were morning-types, 54.5% intermediate-types, and 32.6% evening-types. In line with their diurnal type, weekend sleep midpoint was later among the evening-types – on average 44 min later than among the intermediate-types, and on average 1 h 33 min later than among the morning-types (Table 1). As Table 1 shows, sleep and tiredness on school days varied among the different diurnal types. The evening-types had poorer sleep quality than the other diurnal types. Moreover, the evening-types had shorter sleep duration on school days – on average 14 min less than the intermediate-types and 25 min less than the morning-types. The evening-types had a significantly later bedtime on school days – on average 37 minutes later than the intermediate-types and 1 h 8 min later than the morning-types. Insufficient sleep during the last five school days was more common among the evening-types than the other chronotypes. The evening-types also reported more tiredness on school days than the intermediate- or morning-types. Pearson correlations for sleep variables are presented in Table 2.

**Table 1:** Mean (M), standard deviation (SD), and *p*-values (*p*) of one-way ANOVA tests for age, gender, sleep duration, sleep quality, insufficient sleep, bedtime, and late-night digital media use during school week and chi-square tests for gender and tiredness on school days by diurnal type

	Diurnal type			*p*
	Morning-types M (SD)	Intermediate-types M (SD)	Evening-types M (SD)	
Age	16.94 (0.89)	16.93 (0.92)	16.91 (0.94)	0.933
Gender (female %)	48.2	43.8	47.7	0.412
Sleep and diurnal preference				
Sleep quality	6.21 (4.70)	6.98 (4.77)	8.95 (5.58)	<0.001
Sleep duration on school days	8 h 14 min (56 min)	8 h 3 min (1 h 0 min)	7 h 49 min (1 h 2 min)	<0.001
Bedtime on school days (hh:min)	23:03 (1 h 6 min)	23:34 (1 h 11 min)	00:11 (1 h 9 min)	<0.001
Weekend sleep midpoint (hh:min)	4:49 (1 h 30 min)	5:38 (1 h 19 min)	6:22 (1 h 20 min)	<0.001
Insufficient sleep on school days (in days)	1.81 (1.62)	2.4 (1.99)	3.03 (1.64)	<0.001
Tiredness on school days	4.67 (1.37)	5.73 (1.45)	6.95 (1.44)	<0.001

**Table 2:** Pearson correlations, mean (M) and standard deviation (SD) for sleep variables and age

		1	2	3	4	5	6	M	SD
1	PSQI Sleep quality (poor)							7.51	5.15
2	Sleep duration	−0.04						8 h 0 min	1 h 2 min
3	Bedtime	0.07*	−0.33**					23:42	1 h 14 min
4	Wake-up time	−0.07*	0.74**	0.28**				7:22	58 min
5	Insufficient sleep on school days	0.22**	−0.08**	0.15**	0.01			2.54 days	1.89 days
6	Tiredness on school days	0.39**	−0.10**	0.18**	0.00	0.34**		5.98	1.62
7	Age	0.08**	0.02	0.08*	0.08**	0.00	−0.01	16.93	0.93

*Correlation is significant at 0.05 level, **Correlation is significant at 0.01 level

### Late-Night Digital Media Use by Chronotype

As shown in Table 3, both evening- and intermediate-types, but especially evening-types, used digital media later before going to sleep and reported more negative effects of digital media use than the morning-types. The evening-types also reported higher total use of all media forms and later social media, movies/series or music, gaming, hobbies and studying activities than the other diurnal types. Further, the evening-types experienced more negative effects of digital media use than the other diurnal types. The evening-types also used digital media for longer after lights out than the morning-types or intermediate-types. Although intermediate- and evening-types showed an otherwise similar trend of digital media use to that of morning-types, the other diurnal types’ time used for studying on digital media in the evening did not significantly differ from that of morning-types.

**Table 3:** Frequencies and percentages including adjusted residuals and chi-square tests (*p*) for different forms of evening and late-night media use by diurnal type

Time of evening/night	Diurnal type			*p*
	Morning-types	Intermediate-types	Evening-types	
Total use of all media forms				
Before 9 pm	11 (8%)	16 (2.8%)	8 (2.3%)	<0.001
Adjusted residual	3.2	−1.0	−1.3	
9‒10 pm	37 (26.8%)	101 (17.9%)	32 (9.3%)	
Adjusted residual	3.6	1.6	−4.3	
10‒11 pm	48 (34.8%)	195 (34.6%)	76 (22.2%)	
Adjusted residual	1.2	3.1	−4.1	
11‒12 pm	28 (20.3%)	132 (23.4%)	91 (26.5%)	
Adjusted residual	−1.1	−0.5	1.3	
12‒1 am	3 (2.2%)	73 (13%)	74 (21.6%)	
Adjusted residual	−4.4	−1.4	4.6	
After 1 am	11 (8%)	46 (8.2%)	62 (18.1%)	
Adjusted residual	−1.4	−3.6	4.7	
Social media use				
Before 9 pm	23 (18.1%)	78 (14.7%)	133 (13.6%)	<0.001
Adjusted residual	1.6	1.1	−2.3	
9‒10 pm	37 (29.1%)	110 (20.7%)	187 (19.1%)	
Adjusted residual	3.1	1.4	−3.6	
10‒11 pm	38 (29.9%)	165 (31%)	286 (29.2%)	
Adjusted residual	0.2	1.3	−1.5	
11‒12 pm	23 (18.1%)	103 (19.4%)	197 (20.1%)	
Adjusted residual	−0.6	−0.7	1.1	
12‒1 am	0 (0%)	53 (10%)	115 (11.8%)	
Adjusted residual	−4.4	−1.9	5.2	
After 1 am	6 (4.7%)	23 (4.3%)	60 (6.1%)	
Adjusted residual	−0.7	−2.6	3.2	
Movies/series or music				
Before 9 pm	33 (25.8%)	103 (19.2%)	37 (11%)	<0.001
Adjusted residual	2.7	1.7	−3.7	
9‒10 pm	33 (25.8%)	139 (25.9%)	58 (17.2%)	
Adjusted residual	0.8	2.4	−3.1	
10‒11 pm	38 (29.7%)	143 (26.6%)	84 (24.9%)	
Adjusted residual	0.9	0.1	−0.8	
11‒12 pm	19 (14.8%)	83 (15.5%)	69 (20.5%)	
Adjusted residual	−0.7	−1.5	2.0	
12‒1 am	2 (1.6%)	42 (7.8%)	50 (14.8%)	
Adjusted residual	−3.2	−1.8	4.2	
After 1 am	3 (2.3%)	27 (5%)	39 (11.6%)	
Adjusted residual	−2.2	−2.5	4.2	
Gaming				
Before 9 pm	34 (45.3%)	122 (35%)	60 (30.5%)	0.059
Adjusted residual	2.0	0.1	−1.5	
9‒10 pm	18 (24%)	92 (26.4%)	40 (20.3%)	
Adjusted residual	0.0	1.5	−1.5	
10‒11 pm	13 (17.3%)	74 (21.2%)	40 (20.3%)	
Adjusted residual	−0.7	0.5	−0.1	
11‒12 pm	4 (5.3%)	31 (8.9%)	27 (13.7%)	
Adjusted residual	−1.4	−1.0	2.1	
12‒1 am	2 (2.7%)	16 (4.6%)	12 (6.1%)	
Adjusted residual	−0.9	−0.3	1.0	
After 1 am	4 (5.3%)	14 (4.0%)	18 (9.1%)	
Adjusted residual	−0.2	−2.2	2.4	
Studying				
Before 9 pm	87 (78.4%)	324 (70.4%)	143 (54.6%)	<0.001
Adjusted residual	2.8	2.7	−4.9	
9‒10 pm	18 (16.2%)	81 (23.2%)	69 (26.3%)	
Adjusted residual	−1.1	−2.0	3.0	
10‒11 pm	5 (4.5%)	22 (4.8%)	25 (9.5%)	
Adjusted residual	−0.8	−1.9	2.7	
After 11 pm	1 (0.9%)	33 (7.2%)	25 (9.5%)	
Adjusted residual	−2.7	0.1	1.9	
Hobbies				
Before 9 pm	52 (80%)	170 (62.7%)	92 (63%)	0.065
Adjusted residual	2.7	−1.3	−0.6	
9‒10 pm	9 (13.8%)	63 (23.2%)	25 (17.1%)	
Adjusted residual	−1.4	1.9	−1.1	
10‒11 pm	2 (3.1%)	17 (6.3%)	12 (8.2%)	
Adjusted residual	−1.2	−0.2	1.1	
After 11 pm	2 (3.1%)	21 (7.7%)	17 (11.6%)	
Adjusted residual	−1.6	−0.5	1.8	

As shown in Table 3, morning-types had greater total use of all media forms before 9 pm and from 9 pm to 10 pm but used social media from 9 pm to 10 pm more often than the other diurnal types. They also watched movies/series or listened to music, studied, and participated in hobbies before 9 pm more often than the other diurnal types. The morning-types had greater total use of all media forms from 12 pm to 1 am but used social media after 12 pm less than the other diurnal types. The morning-types also watched movies/series or listened to music more rarely after 12 pm. The intermediate-types had greater total use of all media forms from 11 pm to 12 pm, but watched movies/series or listened to music from 9 pm to 10 pm and studied before 9 pm more often than the other diurnal types. They also had greater total use of all media forms but used social media, watched movies/series, or listened to music and played games after 1 am less often than the other diurnal types The evening-types had greater total use of all media forms from 9 pm to 10 pm but used social media before 9 pm and from 9 to 10 pm less often than the other diurnal types. They also watched movies/series or listened to music less often before 9 pm and from 9 pm to 10 pm and studied less often before 9 pm than the morning- and intermediate-types. The evening-types had greater total use of all media forms, but used social media and watched movies/series or listened to music more often after 12 pm. They also played games more often from 11 pm to 12 pm and after 1 am, and studied more often from 9 pm to 11 pm than the other diurnal types. The percentages of the original sample of time spent using digital media late-night and after lights out are presented in Table 4.

**Table 4:** Percentages of the original sample (*n* = 1127) of time spent using digital media late-night and after lights out

	Time spent using digital media					
	Before 9 pm	9‒10 pm	10‒11 pm	11‒12 pm	12‒1 am	after 1 am
Social media	74.4%	68.8%	56.5%	32.3%	15.2%	6.1%
Movies/series or music	66.5%	64.6%	49.9%	28.6%	14.1%	11.4%
Gaming	48.2%	33.1%	21.6%	10.9%	5.6%	7.0%
Studying	69.1%	22.3%	8.2%	4.3%	1.5%	2%
Hobbies	41.3%	13.9%	5.9%	3.4%	1.5%	1.2%
	Digital media use after lights out					
	Not at all	0‒10 min	10‒20 min	20‒60 min	1‒2 h	over 2 h
	14.7%	17.9%	24.4%	29.1%	9.8%	4.1%

As shown in Table 5, generalized linear regression analysis by weekend midpoint of sleep showed the same effects as diurnal preference. These associations, however, were not as strong for diurnal type as they were for chronotype indicator.

**Table 5:** Time spent using digital media, negative effects of digital media use, and digital media use after lights out by diurnal type (‘morning-type’ as reference group) and weekend sleep midpoint in generalized linear models, adjusted for gender and age

	Evening-type				Intermediate-type				Weekend sleep midpoint			
	B	95% CI		*p*	B	95% CI		*p*	B	95% CI		*p*
Time spent using digital media		Lower	Upper			Lower	Upper			Lower	Upper	
Total use of all media forms	1.51	1.14	1.87	<0.001	0.66	0.32	1.00	<0.001	0.000186	0.000161	0.000209	<0.001
Social media	1.19	0.82	1.56	<0.001	0.46	0.13	0.80	<0.01	0.000145	0.000121	0.000168	<0.001
Movies/series or music	1.16	0.80	1.52	<0.001	0.37	0.03	0.71	<0.05	0.000162	0.000138	0.000185	<0.001
Gaming	0.77	0.28	1.26	<0.01	0.37	−0.09	0.84	0.112	0.000121	0.000092	0.000150	<0.001
Studying	1.11	0.61	1.61	<0.001	0.47	−0.2	0.96	0.058	0.000053	0.000024	0.000082	<0.001
Hobbies	0.91	0.22	1.60	<0.05	0.85	0.21	1.50	<0.05	0.000090	0.000050	0.000130	<0.001
Negative effects of digital media use	0.64	0.47	0.80	<0.001	0.31	0.16	0.46	<0.001	0.000012	0.000002	0.000022	<0.001
Digital media use after lights out	1.12	0.76	1.48	<0.001	0.51	0.18	0.85	<0.01	0.000116	0.000093	0.000138	<0.001

### Late-Night Digital Media Use, Sleep and Tiredness

As shown in Table 6, later digital media use was associated with poorer sleep quality. Those who used social media after 12 pm, or listened to music, watched TV or played games after 1 am had worse sleep quality than those who used social media, listened to music, watched TV or played games before 9 pm. Those who studied using digital media from 9 pm to 10 pm or after 11 pm or took part in hobbies using digital media from 9 to 10 pm had worse sleep quality than those who studied or took part in hobbies using digital media before 9 pm. In addition, 20‒60 min and more than 2 h of digital media use after lights out were associated with worse sleep quality than no digital media use after lights out.

**Table 6:** Sleep quality, sleep duration, bedtime, insufficient sleep, and tiredness on school days by latest hour of digital media use (‘evening media use before 9 pm’ as reference group) and negative effects of digital media use and digital media use after lights out (‘not at all’ as reference group) in generalized linear models, adjusted for gender and age

	PSQI Sleep quality		Sleep duration on school days		Bedtime on school days		Insufficient sleep on school days		Tiredness on school days	
	B	95% CI	B	95% CI	B	95% CI	B	95% CI	B	95% CI
Total use of all media forms										
9‒10 pm	−0.68	[−2.45, 1.09]	−704.63	[−1980.45, 571.18]	11137.14	[−190.62, 2464.90]	−0.27	[−0.69, 0.63]	0.01	[−0.54, 0.56]
10‒11 pm	−0.89	[−2.58, 0.81]	−1431.89*	[−2657.29, −206.49]	2250.85***	[974.72, 3526.97]	0.22	[−0.61, 0.66]	0.31	[−0.21, 0.84]
11‒12 pm	−0.78	[−2.50, 0.95]	−2351.09***	[−3594.41, −1107.77]	4865.05***	[3570.44, 6159.66]	0.19	[−0.45, 0.84]	0.62*	[0.08, 1.15]
12‒1 am	0.51	[−1.28, 2.31]	−3148.51***	[−4443.80, 1853.23]	6579.87***	[5229.17, 7930.58]	0.70*	[0.03, 1.37]	1.16***	[0.60, 1.72]
After 1 am	1.28	[−0.56, 3.13]	−2286.43***	[−3622.18, −950.67]	7512.87***	[6121.24, 8904.49]	0.41	[−0.28, 1.10]	1.14***	[0.56, 1.71]
Social media										
9‒10 pm	0.91	[−0.19, 2.01]	524.62	[−281.04, 1330.28]	−1244.28**	[−2084.64, −403.92]	−0.17	[−0.58, 0.24]	−0.01	[−0.35, 0.34]
10‒11 pm	0.92	[−0.11, 1.94]	−309.37	[−1060.50, 441.77]	185.16	[−598.26, 968.57]	0.40	[−0.34, 0.42]	0.29	[−0.03, 0.61]
11‒12 pm	0.46	[−0.63, 1.56]	−485.67	[−1288.08, 316.74]	2408.21***	[1571.28, 3245.14]	0.14	[−0.27, 0.55]	0.44*	[0.10, 0.78]
12‒1 am	2.17***	[0.93, 3.41]	−1231.97**	[−2142.34, −321.61]	4050.47***	[3098.94, 5001.99]	0.75**	[0.28, 1.21]	1.09***	[0.70, 1.47]
After 1 am	2.43**	[0.91, 3.96]	−1361.75*	[−2492.61, −230.89]	5463.56***	[4294.68, 6632.43]	0.24	[−0.33, 0.81]	1.05***	[0.58, 1.53]
Movies/series or music										
9‒10 pm	−0.07	[−1.05, 0.92]	−348.16	[−1063.03, 366.72]	−61.78	[−845.09, 721.53]	0.36	[−0.00, 0.73]	0.34*	[0.03, 0.65]
10‒11 pm	−0.72	[−1.68, 0.24]	−937.22**	[−1637.02, −237.42]	968.50*	[200.73, 1736.28]	0.22	[−0.14, 0.58]	0.40**	[0.10, 0.71]
11‒12 pm	−0.85	[−1.91, 0.22]	−1328.41***	[−2102.41, −554.42]	3225.08***	[2373.62, 4076.55]	0.46*	[0.07, 0.85]	0.52**	[0.19, 0.86]
12‒1 am	0.68	[−0.59, 1.94]	−1947.19***	[−2868.63, −1025.75]	4715.75***	[3711.95, 5719.56]	0.79***	[0.32, 1.25]	1.13***	[0.73, 1.53]
After 1 am	1.53*	[0.12, 2.93]	−1742.82***	[−2777.81, −707.83]	5539.61***	[4408.49, 6670.74]	0.45	[−0.08, 0.97]	0.84***	[0.39, 1.28]
Gaming										
9‒10 pm	−0.10	[−1.09, 0.89]	43.00	[−743.00, 828.99]	321.53	[−576.60, 1219.66]	−0.13	[−0.54, 0.29]	0.13	[−0.20, 0.45]
10‒11 pm	−0.71	[−1.76, 0.34]	−157.64	[−1001.30, 686.03]	1963.00***	[996.10, 2929.89]	0.01	[−0.42, 0.45]	0.16	[−0.18, 0.51]
11‒12 pm	−0.71	[−2.06, 0.65]	−1264.25*	[−2345.88, −182.62]	4109.47***	[2877.47, 5341.46]	−0.34	[−0.90, 0.23]	0.56*	[0.12, 1.00]
12‒1 am	1.13	[−0.72, 2.98]	−605.93	[−2071.30, 859.44]	4579.98***	[2912.01, 6247.95]	0.76	[−0.02, 1.53]	0.67*	[0.07, 1.27]
After 1 am	2.25*	[0.52, 3.98]	117.32	[−1307.09, 1541.74]	3454.30***	[1810.19, 5098.42]	0.26	[−0.47, 0.98]	1.08***	[0.52, 1.64]
Studying										
9‒10 pm	1.01*	[0.15, 1.86]	−1066.22***	[−1694.79, −437.65]	1040.54 **	[351.04, 1730.04]	0.27	[−0.05, 0.60]	0.27	[−0.01, 0.54]
10‒11 pm	1.03	[−0.37, 2.42]	−1247.13*	[−2266.74, −227.53]	1653.20**	[518.20, 2788.20]	0.24	[−0.29, 0.77]	0.61**	[0.16, 1.05]
After 11 pm	1.93**	[0.57, 3.28]	−2168.01***	[−3170.84, −1165.17]	3896.54***	[2771.39, 5021.69]	0.74**	[0.22, 1.25]	0.89***	[0.46, 1.32]
Hobbies										
9‒10 pm	0.28	[−0.89, 1.45]	−595.49*	[−1450.31, 259.34]	671.45	[−320.55, 1663.44]	0.17	[−0.21, 0.56]	0.16	[−0.22, 0.53]
10‒11 pm	2.09*	[0.19, 3.98]	−1716.92*	[−3112.18, −321.67]	1628.35*	[29.57, 3227.13]	−0.15	[−0.77, 0.47]	0.33	[−0.28, 0.94]
After 11 pm	0.18	[−1.55, 1.91]	−587.28	[−1875.03, 700.47]	3523.34***	[2065.17, 4981.51]	0.25	[−0.33, 0.83]	0.25	[−0.31, 0.80]
Negative effects of digital media use	−1.41***	[1.06, 1.76]	−674.58***	[−935.74, −413.42]	964.18***	[652.90, 1275.47]	0.41***	[0.28, 0.54]	0.55***	[0.44, 0.66]
Digital media use after lights out										
0‒10 minutes	0.34	[−0.72, 1.40]	193.30	[−585.88, 972.49]	808.08	[−97.96, 1714.12]	0.28	[−0.11, 0.66]	0.31	[−0.02, 0.64]
10‒20 minutes	0.32	[−0.67, 1.31]	96.52	[−631.22, 824.26]	1474.48***	[633.14, 2315.83]	0.20	[−0.16, 0.57]	0.42**	[0.11, 0.73]
20‒60 minutes	0.98*	[0.01, 1.94]	−112.81	[−818.57, 592.95]	2296.61***	[1480.96, 3112.26]	0.58**	[0.23, 0.94]	0.77***	[0.47, 1.07]
1‒2 hours	0.81	[−0.43, 2.04]	−36.59	[−945.01, 871.83]	3405.85***	[2357.09, 4454.62]	0.42	[−0.03, 0.88]	0.42*	[0.03, 0.81]
More than 2 hours	1.75*	[0.08, 3.43]	−1658.93**	[−2906.24, −411.62]	4809.11***	[3369.10, 6249.12]	0.72*	[0.10, 1.34]	0.74**	[0.21, 1.26]

*p < 0.05, **p < 0.01, ***p < 0.001

Late-night digital media use was associated with short sleep duration on school days. Those who used any digital media after 10 pm, social media after 12 pm, watched movies/series or listened to music after 10 pm, played games from 11 pm to 12 pm, studied after 9 pm, or took part in hobbies using digital media from 9 pm to 11 pm had shorter sleep duration than those who used the same media before 9 pm. More than 2 h of digital media use after lights out was associated with shorter sleep duration, whereas no use of digital media after lights out was not.

Late-night digital media use was associated with later bedtime. Those who used any digital media after 10 pm, social media from 9 pm to 10 pm or after 11 pm, watched movies/series or listened to music after 10 pm, played games after 10 pm, studied after 9 pm, or took part in hobbies using digital media after 10 pm had a later bedtime than those those who used these media before 9 pm. More than 10 min of digital media use after lights out was associated with a later bedtime, whereas no use of digital media after lights out was not.

Late-night digital media use was associated with insufficient sleep on school days. Those who used any digital media or social media from 12 pm to 1 am, listened to music or watched TV from 11 pm to 1 am, or studied using digital media after 11 pm had insufficient sleep in comparison to those who used these digital media before 9 pm. In addition, 20‒60 min or more than two hours of digital media use after lights out was more often associated with insufficient sleep on school days than no use of digital media after lights out.

Late-night digital media use was also associated with tiredness on school days. Those who used any digital media or social media after 11 pm, listened to music or watched TV after 9 pm, played games after 11 pm, or studied using digital media after 10 pm were more tired than those who used these digital media before 9 pm. In addition, more than 10 min of digital media use after lights out was associated with more tiredness on school days than no use of digital media after lights out.

### Late-Night Digital Media Use as A Mediator of The Relation Between Diurnal Type, Sleep Duration, Sleep Quality, Insufficient Sleep, and Tiredness on School Days

**Fig. 1:**
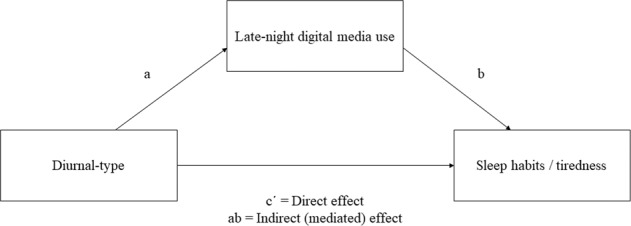
Mediating effect of late-night digital media use on association between diurnal type and sleep/tiredness. Analyses adjusted for gender and participant age. Direct effect (c´) is the association of diurnal type with sleep quality, sleep duration, insufficient sleep, and tiredness, controlling for late-night digital media use. The indirect effect (ab) is the mediated effect

Table 7 presents the results of the mediation analyses. Fig. 1 presents the full mediation model. The significant indirect effects suggest that the total use of all media forms, studying, and the negative effects of digital media use partly mediated the relation between diurnal preference and sleep quality. The total use of all media forms, social media, watching movies or series/listening music, studying and the negative effects of digital media use partly mediated the relation between diurnal preference and sleep duration on school days. The negative effects of digital media use mediated the relation between diurnal preference and insufficient sleep during the five school days. The total use of all media forms, and the negative effects of digital media use mediated the relation between diurnal preference and tiredness on school days.

**Table 7:** Mediation analyses, in which time spent using digital media use mediates the association between sum score for diurnal preference and sleep/tiredness. Analyses adjusted for gender and age. ‘Path a’ is the association between diurnal preference and late-night digital media use, ‘Path b’ is the association between late-night digital media use and sleep/tiredness, and ‘Path c´’ is the direct effect of diurnal preference on sleep/tiredness, controlling for late-night digital media use. ‘Path ab’ is the measure of the mediated effect

	Diurnal preference			
	Path a	Path b	Path c´ = direct effect	Path ab = mediated effect (95% CI)
PSQI Sleep quality				
Social media	−0.09***	0.15	−0.28***	−0.01 (−0.04 to 0.01)
Movies/series or music	−0.10***	0.05	−0.29***	−0.01 (−0.03 to 0.02)
Gaming	−0.06***	0.32*	−0.27***	−0.02 (−0.05 to 0.00)
Studying	−0.05***	0.38*	−0.27***	−0.02 (−0.04 to 0.00)
Hobbies	−0.03*	0.39	−0.32***	−0.01 (−0.04 to 0.00)
Negative effects of digital media use	−0.06***	0.99***	−0.25***	−0.0586 (−0.0863 to −0.0352)
Digital media use after lights out	−0.07***	0.17	−0.30***	−0.0126 (−0.0320 to 0.0027)
Sleep duration on school days				
Total use of all media forms	−0.11***	−493.28***	89.44**	51.67 (29.80 to 77.19)
Social media	−0.09***	−262.50**	124.74***	23.98 (7.14 to 41.89)
Movies/series or music	−0.10***	−358.43***	115.71***	35.98 (17.45 to 59.14)
Gaming	−0.06***	−117.97	123.98**	7.00 (−6.38 to 25.87)
Studying	−0.05***	−588.20***	141.13***	27.00 (12.47 to 45.10)
Hobbies	−0.02*	−362.20	128.81**	8.71 (−0.33 to 30.18)
Negative effects of digital media use	−0.06***	−566.71***	104.20***	33.76 (16.70 to 54.36)
Digital media use after lights out	−0.07***	−49.05	127.39***	3.61 (−9.45 to 18.95)
Insufficient sleep on school days				
Total use of all media forms	−0.11***	0.08	−0.12***	−0.01 (−0.02 to 0.03)
Social media	−0.10***	0.08	−0.12***	−0.01 (−0.02 to 0.00)
Movies/series or music	−0.11***	0.05	−0.13***	−0.01 (−0.01 to 0.00)
Studying	−0.05***	0.11	−0.14***	0.00 (−0.01 to 0.00)
Hobbies	−0.03**	0.03	−0.16***	0.00 (−0.01 to 0.00)
Negative effects of digital media use	−0.06***	0.27***	−0.12***	−0.02 (−0.03 to −0.01)
Digital media use after lights out	−0.08***	0.05	−0.13***	0.00 (−0.01 to 0.00)
Tiredness on school days				
Total use of all media forms	−0.11***	0.15***	−0.21***	−0.02 (−0.03 to −0.01)
Social media	−0.09***	0.11***	−0.21***	−0.01 (−0.02 to 0.00)
Movies/series or music	−0.10***	0.06	−0.22***	−0.01 (−0.01 to 0.00)
Gaming	−0.06***	0.15**	−0.22***	−0.01 (−0.02 to 0.00)
Studying	−0.05***	0.10	−0.23***	−0.01 (−0.01 to 0.00)
Negative effects of digital media use	−0.06***	0.27***	−0.21***	−0.02 (−0.02 to −0.01)
Digital media use after lights out	−0.07***	0.05	−0.22***	−0.00 (−0.01 to 0.00)

*p < 0.05, **p < 0.01, ***p < 0.001

## Discussion

The use of digital media in the evening or at night can delay bedtime and affect sleep quality, which together make young people even more tired during the daytime, resulting in decreased alertness and the capability of learning new things. Previous research has shown a paucity in knowledge on the use of different forms of digital media (e.g., videos, movies, social media), screen content, and their associations with well-being or potential mediating variables (Guerrero et al., 2019). The aspect of late-night digital media use in relation to individual chronotype, which is the focus aspect in the present study, has been studied much less. This study aimed at investigating associations between the use of different forms of digital media and sleep and late-night digital media use mediated the relationship between chronotype and sleep.

The findings presented here provide a deeper insight into the differences between late-night digital media use and sleep among the different chronotypes by revealing that evening-types are at a greater risk of the negative effects of both late-night media use and insufficient sleep. The present findings show that eveningness and late-night digital media use are associated with insufficient sleep and tiredness during the school day. Late-night digital media use partly mediated the relationship between diurnal type and sleep duration, sleep quality, insufficient sleep, and tiredness. The clearest result that emerged from the data is that the associations between adolescents’ late-night media use, insufficient sleep and tiredness were the most pronounced among the evening-types.

In this study, all the different forms of late-night digital media were used the most by the evening-types, especially when assessed by diurnal preference. The most common media used late at night was social media, which poses a higher risk of poor sleep quality for adolescents than the other forms of late-night media use. This finding reflects previous findings that have reported the use of digital media before bedtime as a contributing factor in the current epidemic of insufficient sleep in adolescence (Hysing et al., 2015). The findings of the current study add to the previous research findings that late-night digital media use has a role in this association. What is more, since evening-types might be chronically misaligned in their circadian rhythms, e.g., regarding their actualized sleep-wake behaviour versus their innate sleep-wake phase, they might be at greater risk for the effects of late-night digital media use on quality sleep and alertness.

Adolescents are reported to be especially vulnerable to the effects of phone use and screen time on a good night’s sleep (Quante et al., 2019) and to sleep deprivation in general (Keyes et al., 2015). In this study, eveningness, weekend midpoint, and late-night digital media use were negatively associated with sleep quality and sleep duration on school days and were positively associated with insufficient sleep and tiredness. This finding confirms the results of other studies that have reported that the growing use of digital devices can lead to shorter sleep duration, later bedtimes, more sleep problems (Lemola et al., 2015), and impaired daytime functioning (Hysing et al., 2015).

The current study also confirms previous findings that sleep problems are prevalent among evening-types (Lin et al., 2021; Merikanto et al., 2017; Roeser et al., 2012). Further, the evening-types reported more tiredness on school days than the intermediate- or morning-types. In the general population, eveningness is related to sleep complaints such as insomnia symptoms and having more nightmares (Merikanto et al., 2012). Evening-types are also more vulnerable to adverse health outcomes, for example, behavioural problems (Merikanto et al., 2017), depressive symptoms (Fabbian et al., 2016), and anxiety (Merikanto & Partonen, 2021). Eveningness has also been associated with problematic mobile phone use (Demirhan et al., 2016) and more intensive and intrusive use of common social media applications, such as Facebook (Blachnio et al., 2015), whereas morningness has been negatively associated with both computer and mobile phone usage (Fossum et al., 2014). The findings of the current study are in line with these previous findings, showing that evening-types use different forms of digital media more often and later in the evening. The evening-types also more often reported their digital media use having negative effects than the morning-types. One interesting finding is that the evening-types spent more time on digital media in general and more often used it for social media, movies/series or music, gaming, partaking in hobbies, and studying late in the evening before bedtime than the other diurnal types. Of all the media forms, watching movies/series or listening to music before bedtime were most common among the evening-types.

According to previous research, adolescents delay their bedtime when they are engaged in playing video games and social media (Harbard et al., 2016). Of the different media forms studied here, the evening-types had a higher tendency to watch movies/series or listening to music late at night than the morning-types. As the evening-types tended to watch more movies/series and listen to music in the evening, their later bedtimes eventually led to shorter sleep duration and increased daytime tiredness. The findings of this study confirm that adolescent eveningness, combined with 24/7 access to the internet, predisposes young people to social jet lag by even further delaying the onset of sleep.

The use of social media mediated the association between diurnal type and sleep quality more strongly than the other forms of media use, whereas watching movies or listening to music mediated the connection between diurnal type and sleep duration, insufficient sleep, and daytime tiredness most strongly. Social media use late at night was associated with poor sleep quality, particularly in evening-types. Interestingly, of all the different media forms, studying late at night had the strongest effect on reducing sleep duration and increasing insufficient sleep and daytime tiredness. This finding might be explained by increased stress before bedtime, which makes it more difficult to fall asleep (Knudsen et al., 2007).

As mentioned in the introduction, exposure to blue light, which suppresses the release of melatonin in the evening, increases difficulty falling asleep. Previous studies have also suggested that using a bright display before sleep shortens REM sleep (Higuchi et al., 2005), a sleep phase that is crucial for adolescents’ brain recovery and development, which is associated with the ability to learn new things (Li et al., 2017). The results of the present study suggest that late-night social media use, watching movies/series or listening to music and studying using digital media were associated with later bedtime, shorter sleep duration, tiredness, and decreased sleep quality on school days. Further, the mediation analyses showed that the total use of all media forms – social media, watching movies or listening to music, gaming, and studying late at night with digital media – and the perceived negative effects of digital media use partially mediated the association between diurnal type and sleep or tiredness. These findings emphasize how the mechanisms of chronotype and sleep and tiredness are strongly related to the different forms of digital media use. Although youth between 15 to 20 years of age face different developmental issues, this age range also covers the time period in life with the most drastic circadian change towards eveningness. The current study addresses the interplay between chronotype, late-night media behaviour, sleep and daytime tiredness and offer novel insight on this complex issue during this developmental period of life. However, more research on the associations between chronotype, media use and sleep at different stages of adolescence is needed.

The main strengths of this study were its moderately large survey data and in-depth view of the associations of chronotype, sleep and late-night digital media use with versatile measures of sleep and digital media forms. However, the study also had some limitations. Previous research has suggested that the most accurate results are produced by questions on participants’ sleep over a specific period in recent history, and by measuring sleep duration using the difference between bedtime and wake-up time (Matricciani, 2013). However, sleep duration based on a single school day does not take into account the natural variation in sleep duration on different school days. The scope of this study was limited in terms of the sleep duration reported, which was based on single school days, as this might not necessarily be equivalent to actual sleep duration in the long run. Cronbach’s alpha for tiredness on school days was also slightly low. However, despite these limitations, the study uniquely adds to the understanding of the association between digital media use and sleep. Being the first comprehensive investigation of the associations between chronotype, different forms of digital media use and sleep, its findings have significant implications for understanding how late-night digital media use and sleep vary among different diurnal types.

## Conclusion

The relationship between late-night digital media use and sleep in different chronotypes have remained unknown. In sum, the results presented here uniquely reveal that evening-types are at a greater risk of late-night media use negatively affecting sleep and daytime alertness. Different forms of late-night media use are associated to sleep and well-being in different ways in adolescence. On the question of different forms of late-night media use, this study found that watching movies or listening to music late at night was associated with increased daytime tiredness. In addition, social media use late at night was associated with poor sleep quality. These interactions were pronounced especially for evening-types. Previous findings of intervention studies have shown that limiting social media use can lead to significant improvements in well-being. As late-night digital media use mediates the association between chronotype, sleep and tiredness, these findings suggest that addressing the use of social media, watching movies or listening to music, gaming, and studying with digital media in the evening might prevent sleep problems and enhance well-being in general, especially among evening-types.

## References

[CR1] Åkerstedt T, Nordin M, Alfredsson L, Westerholm P, Kecklund G (2010). Sleep and sleepiness: impact of entering or leaving shiftwork—a prospective study. Chronobiology International.

[CR2] Bartel KA, Gradisar M, Williamson P (2015). Protective and risk factors for adolescent sleep: A meta-analytic review. Sleep Medicine Reviews.

[CR3] Blachnio A, Przepiorka A, Díaz-Morales JF (2015). Facebook use and chronotype: Results of a cross-sectional study. Chronobiology International.

[CR4] Buysse DJ, Hall ML, Strollo PJ, Kamarck TW, Owens J, Lee L, Reis SE, Matthews KA (2008). Relationships between the Pittsburgh Sleep Quality Index (PSQI), Epworth Sleepiness Scale (ESS), and clinical/polysomnographic measures in a community sample. Journal of Clinical Sleep Medicine: JCSM: Official Publication of the American Academy of Sleep Medicine.

[CR5] Buysse DJ, Reynolds CF, Monk TH, Berman SR, Kupfer DJ (1989). The Pittsburgh sleep quality index: A new instrument for psychiatric practice and research. Psychiatry Research.

[CR6] Chaput J-P, Gray CE, Poitras VJ, Carson V, Gruber R, Olds T, Weiss SK, Connor Gorber S, Kho ME, Sampson M, Belanger K, Eryuzlu S, Callender L, Tremblay MS (2016). Systematic review of the relationships between sleep duration and health indicators in school-aged children and youth. Applied Physiology, Nutrition, and Metabolism.

[CR7] Crowley SJ, Cain SW, Burns AC, Acebo C, Carskadon MA (2015). Increased sensitivity of the circadian system to light in early/mid-puberty. The Journal of Clinical Endocrinology & Metabolism.

[CR8] Czeisler CA, Gooley JJ (2007). Sleep and circadian rhythms in humans. Cold Spring Harbor Symposia on Quantitative Biology.

[CR9] de la Vega R, Tomé-Pires C, Solé E, Racine M, Castarlenas E, Jensen MP, Miró J (2015). The Pittsburgh Sleep Quality Index: Validity and factor structure in young people. Psychological Assessment.

[CR10] Demirhan E, Randler C, Horzum MB (2016). Is problematic mobile phone use explained by chronotype and personality?. Chronobiology International.

[CR11] Estevan I, Silva A, Tassino B (2018). School start times matter, eveningness does not. Chronobiology International.

[CR12] Fabbian F, Zucchi B, De Giorgi A, Tiseo R, Boari B, Salmi R, Cappadona R, Gianesini G, Bassi E, Signani F, Raparelli V, Basili S, Manfredini R (2016). Chronotype, gender and general health. Chronobiology International.

[CR13] Fossum IN, Nordnes LT, Storemark SS, Bjorvatn B, Pallesen S (2014). The association between use of electronic media in bed before going to sleep and insomnia symptoms, daytime sleepiness, morningness, and chronotype. Behavioral Sleep Medicine.

[CR14] Giedd JN, Blumenthal J, Jeffries NO, Castellanos FX, Liu H, Zijdenbos A, Paus T, Evans AC, Rapoport JL (1999). Brain development during childhood and adolescence: A longitudinal MRI study. Nature Neuroscience.

[CR15] Guerrero MD, Barnes JD, Chaput J-P, Tremblay MS (2019). Screen time and problem behaviors in children: Exploring the mediating role of sleep duration. International Journal of Behavioral Nutrition and Physical Activity.

[CR16] Hale L, Li X, Hartstein LE, LeBourgeois MK (2019). Media use and sleep in teenagers: What do we know?. Current Sleep Medicine Reports.

[CR17] Hamilton JL, Chand S, Reinhardt L, Ladouceur CD, Silk JS, Moreno M, Franzen PL, Bylsma LM (2020). Social media use predicts later sleep timing and greater sleep variability: An ecological momentary assessment study of youth at high and low familial risk for depression. Journal of Adolescence.

[CR18] Harbard E, Allen NB, Trinder J, Bei B (2016). What’s keeping teenagers up? Prebedtime behaviors and actigraphy-assessed sleep over school and vacation. Journal of Adolescent Health.

[CR19] Hatonen T, Forsblom S, Kieseppa T, Lonnqvist J, Partonen T (2008). Circadian phenotype in patients with the co-morbid alcohol use and bipolar disorders. Alcohol and Alcoholism.

[CR20] Hayes, A. F. (2018). *Introduction to mediation, moderation, and conditional process analysis: A regression-based approach* (Second edition). Guilford Press.

[CR21] Higuchi S, Motohashi Y, Liu Y, Maeda A (2005). Effects of playing a computer game using a bright display on presleep physiological variables, sleep latency, slow wave sleep and REM sleep. Journal of Sleep Research.

[CR22] Hysing M, Harvey AG, Linton SJ, Askeland KG, Sivertsen B (2016). Sleep and academic performance in later adolescence: Results from a large population-based study. Journal of Sleep Research.

[CR23] Hysing M, Pallesen S, Stormark KM, Jakobsen R, Lundervold AJ, Sivertsen B (2015). Sleep and use of electronic devices in adolescence: Results from a large population-based study. BMJ Open.

[CR24] Jones, S. E., Lane, J. M., Wood, A. R., van Hees, V. T., Tyrrell, J., Beaumont, R. N., Jeffries, A. R., Dashti, H. S., Hillsdon, M., Ruth, K. S., Tuke, M. A., Yaghootkar, H., Sharp, S. A., Jie, Y., Thompson, W. D., Harrison, J. W., Dawes, A., Byrne, E. M., Tiemeier, H., … Weedon, M. N. (2019). Genome-wide association analyses of chronotype in 697,828 individuals provides insights into circadian rhythms. *Nature Communications*, *10*(1). 10.1038/s41467-018-08259-7. 10.1038/s41467-018-08259-7 PMC6351539 30696823

[CR25] Keyes KM, Maslowsky J, Hamilton A, Schulenberg J (2015). The Great Sleep Recession: Changes in Sleep Duration Among US Adolescents, 1991-2012. PEDIATRICS.

[CR26] Knudsen HK, Ducharme LJ, Roman PM (2007). Job stress and poor sleep quality: Data from an American sample of full-time workers. Social Science & Medicine.

[CR27] Koskenvuo M, Hublin C, Partinen M, Heikkilä K, Kaprio J (2007). Heritability of diurnal type: A nationwide study of 8753 adult twin pairs. Journal of Sleep Research.

[CR28] Kronholm E, Puusniekka R, Jokela J, Villberg J, Urrila AS, Paunio T, Välimaa R, Tynjälä J (2015). Trends in self-reported sleep problems, tiredness and related school performance among Finnish adolescents from 1984 to 2011. Journal of Sleep Research.

[CR29] Kuula L, Pesonen A-K, Martikainen S, Kajantie E, Lahti J, Strandberg T, Tuovinen S, Heinonen K, Pyhälä R, Lahti M, Räikkönen K (2015). Poor sleep and neurocognitive function in early adolescence. Sleep Medicine.

[CR30] Lemola S, Perkinson-Gloor N, Brand S, Dewald-Kaufmann JF, Grob A (2015). Adolescents’ Electronic Media Use at Night, Sleep Disturbance, and Depressive Symptoms in the Smartphone Age. Journal of Youth and Adolescence.

[CR31] Li W, Ma L, Yang G, Gan W-B (2017). REM sleep selectively prunes and maintains new synapses in development and learning. Nature Neuroscience.

[CR32] Lin, C., Imani, V., Griffiths, M. D., Broström, A., Nygårdh, A., Demetrovics, Z., & Pakpour, A. H. (2021). Temporal associations between morningness/eveningness, problematic social media use, psychological distress and daytime sleepiness: Mediated roles of sleep quality and insomnia among young adults. *Journal of Sleep Research*, *30*(1). 10.1111/jsr.13076. 10.1111/jsr.13076 32406567

[CR33] Matricciani L (2013). Subjective reports of children’s sleep duration: Does the question matter? A literature review. Sleep Medicine.

[CR34] Merikanto, I., Kantojärvi, K., Partonen, T., Pesonen, A.-K., & Paunio, T. (2021). Genetic variants for morningness in relation to habitual sleep-wake behavior and diurnal preference in a population-based sample of 17,243 adults. *Sleep Medicine*, S138994572100071X. 10.1016/j.sleep.2021.01.054. 10.1016/j.sleep.2021.01.054 33631501

[CR35] Merikanto I, Kronholm E, Peltonen M, Laatikainen T, Lahti T, Partonen T (2012). Relation of chronotype to sleep complaints in the general finnish population. Chronobiology International.

[CR36] Merikanto I, Lahti J, Kuula L, Heinonen K, Räikkönen K, Andersson S, Strandberg T, Pesonen A-K (2018). Circadian preference and sleep timing from childhood to adolescence in relation to genetic variants from a genome-wide association study. Sleep Medicine.

[CR37] Merikanto I, Partonen T (2021). Eveningness increases risks for depressive and anxiety symptoms and hospital treatments mediated by insufficient sleep in a population‐based study of 18,039 adults. Depression and Anxiety.

[CR38] Merikanto I, Pesonen A-K, Kuula L, Lahti J, Heinonen K, Kajantie E, Räikkönen K (2017). Eveningness as a risk for behavioral problems in late adolescence. Chronobiology International.

[CR39] Michaud I, Chaput J-P (2016). Are Canadian children and adolescents sleep deprived. Public Health.

[CR40] Mireku MO, Barker MM, Mutz J, Dumontheil I, Thomas MSC, Röösli M, Elliott P, Toledano MB (2019). Night-time screen-based media device use and adolescents’ sleep and health-related quality of life. Environment International.

[CR41] Poulain T, Vogel M, Buzek T, Genuneit J, Hiemisch A, Kiess W (2019). Reciprocal longitudinal associations between adolescents’ media consumption and sleep. Behavioral Sleep Medicine.

[CR42] Prensky M (2001). Digital Natives, Digital Immigrants Part 2: Do They Really Think Differently?. On the Horizon.

[CR43] Quante M, Khandpur N, Kontos EZ, Bakker JP, Owens JA, Redline S (2019). “Let’s talk about sleep”: A qualitative examination of levers for promoting healthy sleep among sleep-deprived vulnerable adolescents. Sleep Medicine.

[CR44] Rafique N, Al-Asoom LI, Al Sunni A, Saudagar FN, Almulhim LA, Alkaltham GK (2020). Effects of mobile use on subjective sleep quality. Nature and Science of Sleep.

[CR45] Reidy BL, Raposa EB, Brennan PA, Hammen CL, Najman JM, Johnson KC (2016). Prospective associations between chronic youth sleep problems and young adult health. Sleep Health.

[CR46] Roenneberg T, Daan S, Merrow M (2003). The art of entrainment. Journal of Biological Rhythms.

[CR47] Roenneberg T, Kuehnle T, Juda M, Kantermann T, Allebrandt K, Gordijn M, Merrow M (2007). Epidemiology of the human circadian clock. Sleep Medicine Reviews.

[CR48] Roeser K, Brückner D, Schwerdtle B, Schlarb AA, Kübler A (2012). Health-related quality of life in adolescent chronotypes—a model for the effects of sleep problems, sleep-related cognitions, and self-efficacy. Chronobiology International.

[CR49] Scott H, Woods HC (2019). Understanding links between social media use, sleep and mental health: recent progress and current challenges. Current Sleep Medicine Reports.

[CR50] Tarokh L, Short M, Crowley SJ, Fontanellaz-Castiglione CEG, Carskadon MA (2019). Sleep and circadian rhythms in adolescence. Current Sleep Medicine Reports.

[CR52] van der Schuur WA, Baumgartner SE, Sumter SR (2019). Social media use, social media stress, and sleep: examining cross-sectional and longitudinal relationships in adolescents. Health Communication.

[CR53] Horne JA, Östberg O (1976). A self-assessment questionnaire to determine morningness-eveningness in human circadian rhythms. International Journal of Chronobiology.

